# Improving Documentation of Postdischarge Issue Mitigation during Postdischarge Phone Calls

**DOI:** 10.1097/pq9.0000000000000618

**Published:** 2022-11-10

**Authors:** Sarah Vepraskas, Snezana Nena Osorio, Sarah Corey Bauer, Alyssa Stephany, Sandra Gage

**Affiliations:** From the * Department of Pediatrics, Medical College of Wisconsin, Children’s Hospital of Wisconsin, Milwaukee, Wisconsin; † Department of Pediatrics, Weill Cornell Medicine, The New York Presbyterian Hospital, New York, New York; ‡ Department of Pediatrics, Children’s Mercy Kansas City, University of Missouri-Kansas City, Kansas City, Missouri; § Department of Pediatrics, Phoenix Children’s Hospital, University of Arizona, Phoenix, Arizona.

## Abstract

Postdischarge phone calls (PDPCs) are recommended to identify and mitigate postdischarge issues such as missed follow-up appointments, medication errors, and failure to activate contingency plans. A current state assessment showed variability in documenting PDPC content and postdischarge issue mitigation. Therefore, the primary aim was to improve the postdischarge issue mitigation documentation rate from 65% to 100% over 16 months.

**Methods:**

An interdisciplinary quality improvement team used the Model for Improvement to perform planned sequential interventions over 16 months. The outcome measure was documentation of postdischarge issue mitigation. Process measures included PDPC template use and completion and postdischarge issue identification. Balancing measures included call attempts and caller perceptions of ease of documentation. Interventions included creating a flowsheet note template, creating caller template training sessions, and sharing team data and feedback. We gathered data via reports generated from the electronic medical record, chart review, and survey. Data were analyzed using statistical process control charts and established rules for detecting special cause variation.

**Results:**

The postdischarge issue mitigation documentation increased from 65% to 91% over 16 months. Template use and completion increased from 0% to 100% and 98%, respectively. The number of postdischarge issues identified remained unchanged. Call attempts increased from 40% to 59%. Caller perceptions remained unchanged.

**Conclusions:**

Documentation of postdischarge issues and issue mitigation promotes adequate communication with the patient’s care providers, improving the quality and safety of care. Data sharing to promote team engagement was the key factor in improving documentation of postdischarge issue mitigation.

## INTRODUCTION

Poor understanding of the discharge plan for pediatric patients poses safety risks. It can lead to postdischarge issues such as missed follow-up appointments, medication errors, and failure to activate contingency plans.^1–5^ To mitigate these safety risks, many evidence-based and patient-centered discharge transition bundles have been developed, with most supporting the use of postdischarge phone calls (PDPCs) as adjunct safety tools and a component of complete hospital-to-home care.^6–9^ Several studies have demonstrated the potential benefit of the PDPC in the early detection of postdischarge issues.^1,2,4,10,11^ Two studies of PDPCs in children detected an alarming percentage of postdischarge issues following release from a medical service.^2,4^ The largest retrospective analysis of hospital-initiated follow-up contact of pediatric patients to date demonstrated that 25% of patients experience postdischarge issues, with approximately 76% of identified issues related to follow-up appointments and 21% due to medication issues.^4^

Although there is no gold standard for pediatric PDPC content, the Agency for Health Care Research and Quality Re-Engineered Discharge tool kit used evidence to develop the 5 key components of a PDPC, which include: assessment of health status, medication review, clarification of clinician appointments and scheduled studies, coordination of postdischarge home services, and review of the discharge contingency plan.^8^ These elements align with the most common postdischarge issues noted in the literature.^2,4,12,13^ However, a standardized format does not exist for documenting PDPCs in the electronic health record (EHR). Therefore, postdischarge issues and actions taken for mitigation are not always captured. Careful documentation of the issue(s) identified and what was done to address the issue is essential to ensuring adequate provider communication, especially if medications or instructions are altered.

We conducted a current state assessment of programs and units performing PDPCs at our hospital, revealing that only 1 inpatient unit, the neuroscience unit, consistently performed PDPCs focusing on patient satisfaction. In addition, we reviewed the telephone encounters from the PDPCs, which revealed inconsistent documentation of issues identified and mitigation efforts. With these inconsistencies noted, the team sought to improve PDPC content, with a global aim to utilize PDPCs to improve patient safety during the transition of care. Our primary aim was to improve the postdischarge issue mitigation documentation rate from 65% to 100% over 16 months.

## METHODS

### Context

#### Institution and Unit Description

We performed our study at Children’s Wisconsin, a free-standing tertiary care pediatric hospital with 306 beds, 16,321 admissions per year, and an average daily census of 208 patients. Our EHR is Epic (Verona, WI). We initiated the pilot study in the 22-bed neuroscience unit. Patients discharged from this unit are often medically complex and receive multiple medications, representing a vulnerable population. The following specialists manage patients on this unit: neurosurgery, epileptology, and pediatric hospital medicine, many with neurology consultation. The average daily census is 15. Also, this unit has dedicated nurse callers, and the PDPC processes to assess patient satisfaction were already in place.

#### Population Description and PDPC Workflow

We included patients discharged from the neuroscience unit, including those with non-English-speaking caregivers and patients in foster care. We excluded patients who were followed by the complex care program (they received a PDPC from the complex care nurses) and those who were transferred to an inpatient behavioral health or rehabilitation facility. The health unit coordinator kept a copy of the patients discharged daily, and the nurses reviewed the list and identified eligible patients. The nurse callers contacted eligible patients 24–96 hours after discharge. Due to time constraints, the caller usually performs 1 PDPC attempt per patient. Before the PDPC note template development, the nurse callers asked caregivers questions focused on patient experience and used the responses to drive unit-level improvements. Nurse callers completed documentation via free text in an EHR telephone encounter note without a structured note template.

### Planning the Interventions

**Fig. 1.**
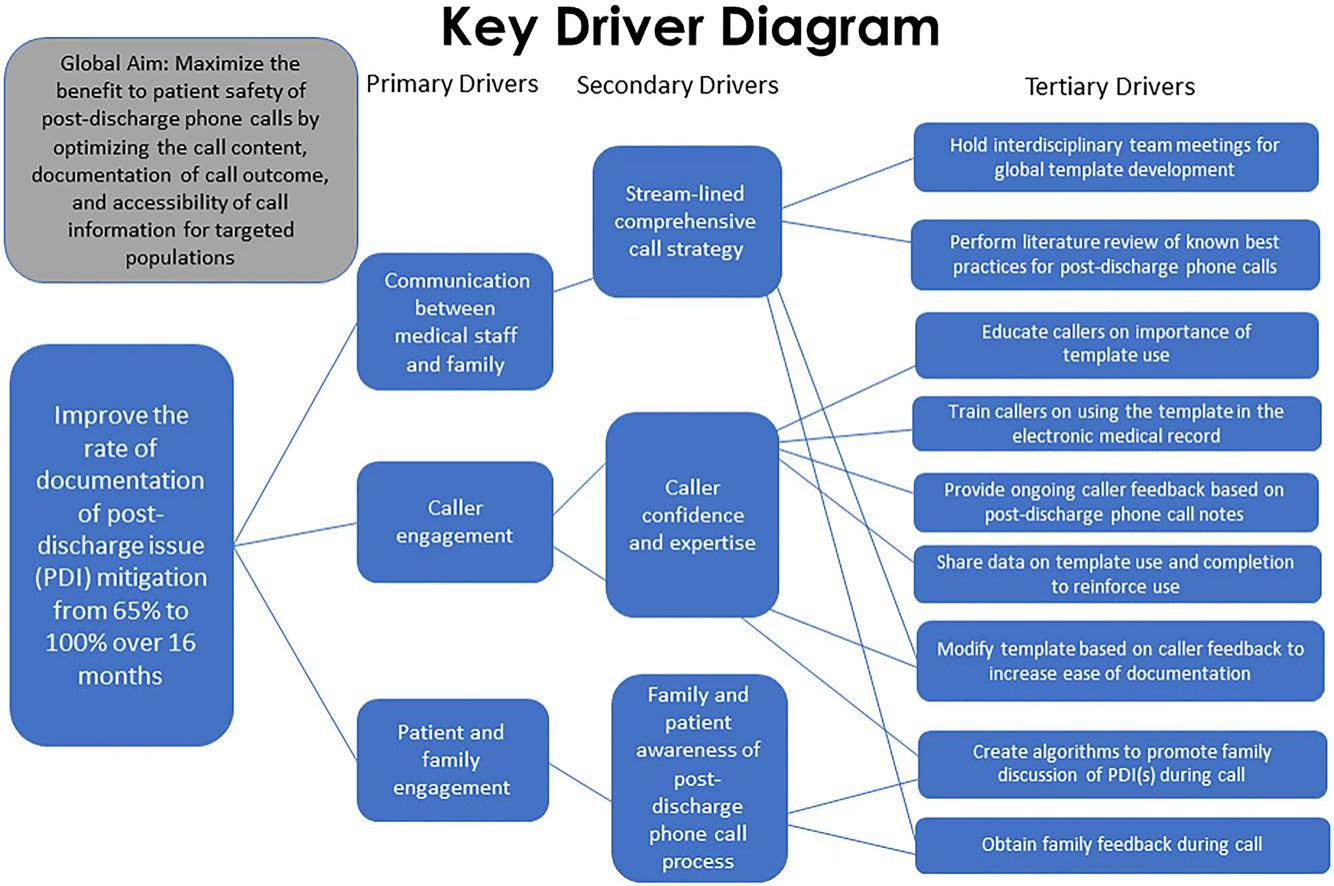
Key driver diagram for improving the rate of documentation of postdischarge issue mitigation.

We assembled an interprofessional quality improvement team, including the neuroscience unit leadership, neuroscience nurses performing the PDPC, hospital medicine attendings, health literacy experts, family advisory council representatives, and EHR analysts. Through group collaboration, we developed strategies to improve the rate of postdischarge issue mitigation. Key drivers included (1) a streamlined, comprehensive call strategy, (2) designated caller expertise and education, and (3) patient and family engagement (Fig. 1). We created a PDPC note template using available literature and an iterative process tailored to best fit our population and address a streamlined call strategy. We held note template training sessions to improve caller expertise and education and provided ongoing caller feedback. Patient and family perceptions were obtained at the end of the call via query regarding call efficacy, prompting discussion of any undiscovered issues (tertiary drivers, Fig. 1).

### Interventions

#### Determination of Call Content

**Fig. 2.**
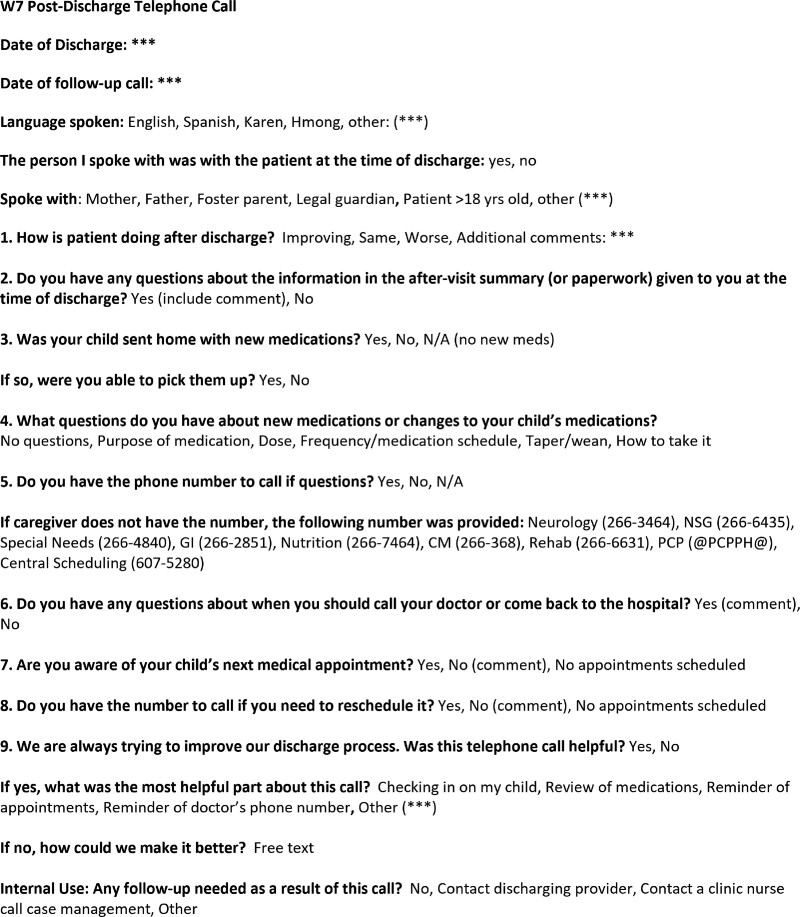
PDPC Questions (in bold), with answer options after the questions. Callers could pick more than 1 option for question 4. Asterisks denote free-text functionality.

We modified the previous phone call content to include the essential safety elements of a PDPC, as recommended by the Agency for Health Care Research and Quality Re-Engineered Discharge tool kit.^8^ After an iterative process to modify the PDPC note template, we considered 4 out of the 5 elements most relevant for our population, including health status, medication review, clarification of clinician appointments and scheduled studies, and review of the discharge contingency plan. We finalized the questions via workgroup consensus (Fig. 2), and our institution’s health literacy committee ensured family-friendly language at the fifth-grade level. In addition, we included a free-text option for callers to promote documentation of actions taken if a postdischarge issue was discovered.

#### Development of EHR Pop-up Flowsheet and PDPC Note Template

**Fig. 3.**
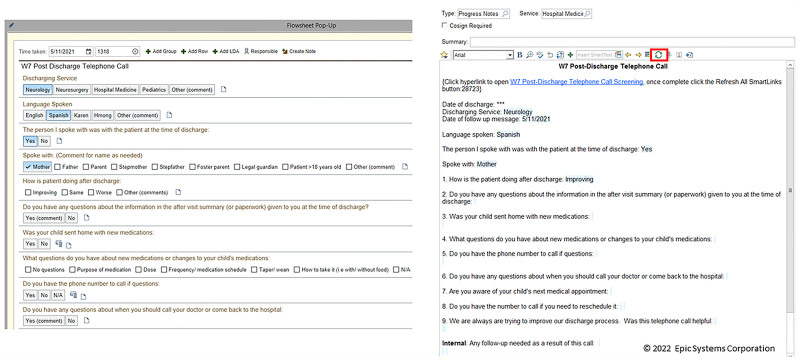
EHR pop-up flowsheet and PDPC note template. Once fields were selected (left) and the flowsheet pop-up was completed, nurse callers would click the refresh button (highlighted in red above left) to autopopulate the components of the pop-up flowsheet into the note template. Anonymous electronic survey given to callers (in bold), with answer options after the questions. A baseline survey was given before template implementation and then quarterly for 9 months following template implementation.

Based on the previous literature supporting pop-up flowsheets to provide easily accessible data in the EHR,^14^ we created a PDPC EHR pop-up flowsheet embedded in a note template for the nurses to efficiently document caller responses. After completing the pop-up flowsheet, the nurse could select the refresh button to autopopulate the responses into a note template (Fig. 3).

#### PDPC Caller Training Program and Template Training Sessions

We implemented a caller-training program that included scripting for callers and a resource guide for postdischarge issues commonly identified in the neuroscience population. The resource guide includes clinic numbers for neuroscience clinics and how to contact other medical specialties. In addition, we trained callers to use the EHR note template via group sessions and individual discussions for unique scenarios.

#### Team Data Sharing and Feedback

**Fig. 4.**
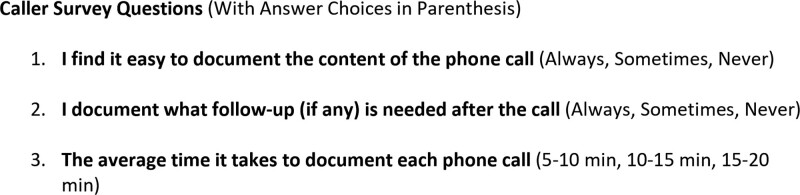
Anonymous electronic survey given to callers (in bold), with answer options after the questions. A baseline survey was given before template implementation and then quarterly for 9 months following template implementation.

The team leader reviewed the responses and documentation from the PDPC weekly. Moreover, we held quarterly meetings with the nurse callers and unit leadership team to discuss postdischarge issues and share examples of excellent documentation, troubleshoot barriers, and reinforce favored strategies. Also, the nurse callers completed a baseline survey, including questions regarding ease and time of call documentation. The survey was repeated quarterly for 9 months following implementation (Fig. 4).

### Study of the Interventions

We performed a retrospective chart review to gather baseline data on PDPC content for 3 months before implementing our EHR-based PDPC note template. Following note template implementation, we generated a PDPC summary report from the EHR via flowsheet data. We obtained phone call attempts and the completion rate by reviewing the shared unit-level PDPC spreadsheet. We accessed completed survey data through Qualtrics electronic survey platform.^15^

### Measures

Our primary outcome measure was the documentation rate of postdischarge issue mitigation per month (ie, the number of issues with documentation of mitigation/the number of issues identified). We determined postdischarge issue mitigation using the clinical data report and chart review to assess postdischarge issues and postdischarge issue mitigation documentation. Process measures included PDPC note template use and completion and identification of postdischarge issues. Balancing measures included phone call attempts and caller perceptions of ease of documentation.

### Analysis

Shewhart P-charts were used to plot and analyze data. We analyzed our initial means based on data collected 3 months before our initial intervention. We used the Shewhart chart rules to identify special cause variation leading to any shifts in the mean.^16^ We calculated the centerline (CL) (mean) and upper and lower control limits using QI Charts (licensed by Richard Scoville, Scoville Associates, 2009).^16^ We analyzed survey data using comparative statistics, observing trends in reported perceptions.

### Ethical Considerations

Our hospital’s institutional review board reviewed this project and deemed its quality improvement, not human subject research.

## RESULTS

During the 3-month preintervention period, we collected baseline data from 75 PDPCs, and 10 (13%) issues were identified, with 8 out of 10 (80%) having mitigation documentation. Three out of 10 (30%) postdischarge issues were medication-related. From January 2019 to March 2020 (intervention period), the nurse callers made 591 PDPCs, and 81 issues were identified, with 67 (83%) having mitigation documentation. Forty-six percent (37/81) of issues identified were medication-related, such as inconsistencies between medication dosages documented on the after-visit summary and what the pharmacy had on file.

### Outcome Measure

**Fig. 5.**
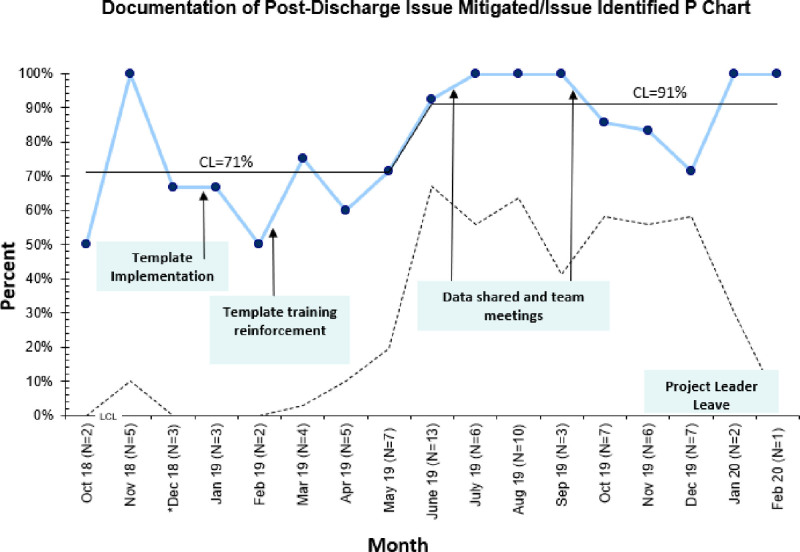
Statistical process control chart demonstrating percent of charts with documentation of postdischarge issue mitigation by month. UCL not shown when calculated more than 100%. UCL, lower control limit. (Mean shifted using the rule of having 8 or more consecutive points above the CL.) *The outlier noted during the baseline period was removed.

Documentation of postdischarge issue mitigation increased from 71% to 91% over 16 months (Fig. 5). Key interventions to achieve improvement included note template training and reinforcement with regular data sharing and team meetings. In addition, we shared examples of ideal documentation at team meetings to promote thorough documentation.

### Process Measures

The nurse callers integrated the note template into their workflow, and as a result, template use went from 0% to 100% during the intervention period. Also, the note template completion increased from 0% to 98%. There was no change in the percentage of postdischarge issues identified during the calls from the preintervention to the intervention period, with a range of 3%–29% per month with no clear trend.

### Balancing Measures

**Fig. 6.**
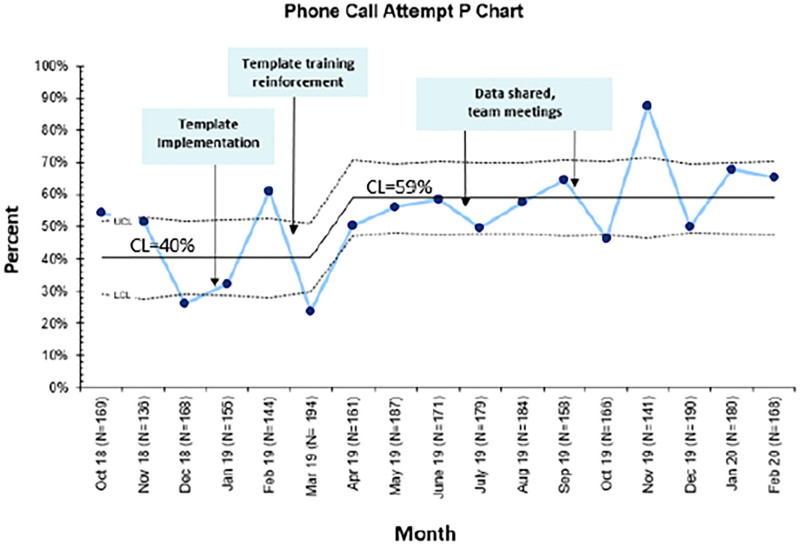
Statistical process control chart demonstrating percent of phone call attempts by month. (Mean shifted using the rule of having 8 or more consecutive points above the CL).

During the intervention period, rather than observing a decrease in phone call attempts to discharged patients, we observed an increase in the mean number of phone call attempts from 40% to 59% of discharged patients (Fig. 6). The connectivity rate (ie, completed phone calls/phone call attempts) remained stable at approximately 45%. Survey reports of caller perceptions of time and ease of documentation were unchanged over the 9 months of evaluation. The response rate to the anonymous caller surveys varied from 50% to 75% per cycle.

## DISCUSSION

We increased the postdischarge issue mitigation documentation rate through sequential interventions from 71% to 91% over 16 months. Our results are consistent with the literature demonstrating that approximately 25% of children discharged from the hospital experience postdischarge issues.^2,4^ In concordance with the previous studies, the most common postdischarge issues identified and mitigated were medication-related.^2,4,12,13^ The identification and mitigation of postdischarge issues are a protective measure against the safety risks at discharge. Furthermore, documentation of these issues and how the issues were mitigated promote adequate communication with the patients’ care providers. The note template was universally used following development because it streamlined call content documentation, including mitigation of postdischarge issues. Importantly, the callers did not perceive that the template took more time to use. Postdischarge issues and phone call connectivity remained stable before and after note template implementation. In contrast, the percentage of call attempts and mitigation documentation increased, which we postulate was due to the implementation of our template. Therefore, the callers reached more patient families, and more issues were mitigated, supporting the effectiveness of the note template.

In our initiative, caller training and staff engagement were critical in improving documentation of postdischarge issue mitigation. The key intervention was data sharing during team meetings to promote team engagement and expertise. By reviewing the data and discussing examples from previous calls, the nurse callers became more comfortable with the mitigation process and the relevant detail necessary for documentation. However, engagement declined without frequent reminders, indicating further work is required to sustain this change in the system. This theory is reinforced by a 3-month decrease in postdischarge issue mitigation documentation, with 2 of these months occurring during a leave taken by the study’s leader. During this period, data sharing and feedback sessions were lacking. To promote sustainability, our next steps may be to use an EHR forcing function, making it mandatory to document the mitigation steps if a postdischarge issue is recorded.

As previously mentioned, our study’s limitations include the lack of sustainability of postdischarge issue mitigation documentation. The importance of the documentation of the postdischarge issue and issue mitigation required constant reminders, likely because the safety component of the call was a new addition to the unit’s PDPC program. Another limitation included the inconsistent response rate to our anonymous nurse surveys and the concern that they may not have captured all perspectives of the nurse callers. However, since we saw improvements in postdischarge issue mitigation and phone call attempts, we believe the surveys captured most of the callers’ viewpoints.

The next steps for our workgroup are to consider ways to promote sustainability and to share our PDPC note template with other acute care units or programs in our institution. Although providers must focus on safe transitions of care to prevent postdischarge issues, unfortunately, they still happen. Thus, emphasis must be placed on documentation of postdischarge issue mitigation, allowing the healthcare team members to understand the patient issue(s) and mitigation that occurred. Given that we did demonstrate an increase in documentation of the mitigation of postdischarge issues, other institutions may consider implementing a PDPC program and using a template to improve postdischarge issue documentation and mitigation. This practice will continue to improve our patients’ care quality and safety.

## ACKNOWLEDGMENT

Assistance with the study: Thank you to the neuroscience unit leadership and the nurse callers for assisting with the study and the caregivers and parents who engaged in the postdischarge phone calls.

## DISCLOSURE

The authors have no financial interest to declare in relation to the content of this article.
